# Prevention of liver tumor formation in woodchucks with established hepatocellular carcinoma by treatment with cationic liposome-DNA complexes

**DOI:** 10.1186/s12885-017-3163-2

**Published:** 2017-03-06

**Authors:** Jeffery Fairman, Katherine H. Liu, Stephan Menne

**Affiliations:** 1 Juvaris BioTherapeutics, Inc., Pleasanton, CA 94566 USA; 2 Present address: SutroVax, Inc., South San Francisco, CA 94080 USA; 3 Department of Clinical Sciences, College of Veterinary Medicine, Cornell University, Ithaca, NY 14853 USA; 4 Present address: Georgetown University Medical Center, Department of Microbiology & Immunology, Medical-Dental Building, Room C301, 3900 Reservoir Road, Washington, DC 20057 USA

**Keywords:** Cationic liposome–DNA complexes, Hepatitis B virus, Hepatocellular carcinoma, Woodchuck, Immunotherapy

## Abstract

**Background:**

Approximately 250 million people worldwide are chronically infected with hepatitis B virus (HBV) and more than half of the hepatocellular carcinoma (HCC) cases are attributed to this infection. As HCC has a high mortality rate, and current treatment options are remarkably limited, the development of new therapeutic treatment strategies is warranted.

**Methods:**

In this study, woodchucks infected with woodchuck hepatitis virus (WHV), and with pre-existing liver tumors, were used as a model to investigate if complexes of cationic liposomes and non-coding DNA (JVRS-100) were effective in treatment of HCC.

**Results:**

It was observed that the high serum viral load that is present in a typical chronic WHV infection (i.e., approximately 100-fold higher than human viral loads) results in immune suppression and resistance to treatment with JVRS-100. Treatment of woodchucks with lower serum viral load that more closely matched with the viral load usually seen in human HBV infection appears a better model for immunotherapeutic development based on the responsiveness to JVRS-100 treatment. In the latter case, marked declines in WHV DNA and WHV surface antigen were determined over the 12-week treatment period and WHV markers stayed suppressed during most time points of the 12-week follow-up period. Even more remarkably, the formation of new liver tumors was not observed in woodchucks treated with a well-tolerated dose of JVRS-100, as compared to several new tumors that developed in vehicle-treated control animals.

**Conclusions:**

Although there was little decrease in the volumes of the liver tumors existing at the time of treatment, it is generally accepted that preventing the spread and metastasis of almost always fatal cancers such as HCC and thus, reducing it to a chronic and treatable disease can also be a successful therapeutic approach. The results in woodchucks warrant the investigation of JVRS-100 as an intervention to prevent liver cancer in patients chronically infected with HBV and at high risk for HCC development.

## Background

Chronic infection with hepatitis B virus (HBV) is a major cause of hepatocellular carcinoma (HCC), which is the fifth most common cancer in the world and the third leading cause of cancer deaths [1, 2]. HCC has a high mortality rate because it is frequently asymptomatic and the patient does not seek medical attention until it is too late for surgical removal [2]. Treatment options are limited as HCC at an advanced stage does not respond well to chemotherapy [2, 3]. Therefore, there is an urgent need for developing new treatment strategies against HCC, in general and against HBV-induced HCC, in particular.

Infection with HBV is a major public health problem and is responsible for an estimated 1.2 million deaths per year worldwide. Death is attributed in most cases to the development of chronic liver injury, cirrhosis, and primary HCC. Estimates are that more than 2 billion people throughout the world have serological evidence of previous or current HBV infection and that at least 248 million individuals are chronic carriers of HBV [4, 5]. There are believed to be at least one million carriers in the United States alone [6, 7]. Approved treatment strategies for persistent HBV infection include the use of (pegylated) interferon-alpha and several nucleos(t)ide analogs, such as lamivudine, adefovir, tenofovir, telbivudine, and entecavir. Less than 30% of the patients, however, have sustained antiviral response and adverse side-effects are significant [8–10]. Furthermore, the use of current antiviral drugs is limited due to the emergence of drug resistant variants and the risk of relapse upon treatment discontinuation. Although there is an efficacious prophylactic HBV vaccine, and recent studies have shown that vaccination of infants significantly reduces the development of liver cancer [11], chronic HBV infections are on the rise as well as an alarming increase in the incidence of liver cancer in the United States. As a result, liver cancer is fast becoming an increasing public health threat in the United States and has a five-year survival rate of less than 10%, making it one of the deadliest cancers in this country.

While antiviral treatments are able to keep viral load at low or undetectable levels, recent studies have shown that successfully treated patients still exhibit significant levels of HBV-induced liver disease above those in uninfected individuals and that the risk of liver cancer is not eliminated even in this cohort of patients [12]. Although infected patients with low or undetectable viral load in their blood system are less likely to develop liver cancer, compared to patients with detectable viral load, these individuals are still at a much higher risk than is the general uninfected population [13]. Furthermore, the emergence of HBV strains which are not effectively neutralized by the current vaccine is also a significant problem [14, 15].

There have been recent advances in the treatment of HCC, such as the approval of Sorafenib, a small molecule receptor inhibitor of several tyrosine and Raf kinases. However, treatment benefit is modest as only an approximately three month improvement in overall survival is achieved [16]. Therefore, continued development of therapeutic approaches to control HBV infection and HCC occurrence is highly warranted and an unmet medical need.

Complexes consisting of cationic/neutral lipid carrier and non-coding DNA plasmid (CLDC; referred to here as JVRS-100) are potent stimulants of innate immunity [17–19]. Stimulation by JVRS-100 is mainly due to a liposome-mediated potentiation of the mammalian innate immune response to non-methylated CpG motifs within the plasmid. Cationic liposomes facilitate endocytosis and direct delivery of the plasmid DNA to the endosomal compartment of cells. The targeted delivery results then in increased binding of nucleic acids to endosomally located toll-like receptors (TLRs), including TRL9, TLR7/8, and TLR3 molecules thereby leading to enhanced innate immune activation. Furthermore, in vivo evidence suggests that the induction of a strong T_H_1-type immune response is based in part by activation of cytolytic T lymphocytes (CTLs) and natural killer (NK) cells and production of interleukin (IL) 12 (IL-12) and type I and II interferons [20–22] all of which are known to be important mediators of antitumor immunity. JVRS-100 administered in combination with tumor cell lysates has efficacy against tumor progression in mouse models of cancer and in dogs with naturally-occurring tumors and increased survival in these animal models [23–25]. Furthermore, the combination of JVRS-100 with antigen resulted in a potent adjuvant effect as well as in robust antibody and CTL responses to the target antigen. Based on these results, the evaluation of antitumor effects of JVRS-100 in a fully immunocompetent animal model of chronic HBV infection with virus-induced HCC is warranted.

The woodchuck model of chronic HBV infection is recognized as a valuable translational animal model for HBV-related research [26]. The Eastern woodchuck (*Marmota monax*) infected with the woodchuck hepatitis virus (WHV) has been used for studies of the pathogenesis of chronic HBV infection and in the preclinical evaluation of efficacy and safety of antiviral compounds for the prevention of HBV disease sequelae, including HCC. This animal model mimics many of the virological and immunological response features observed in human HBV infection [26] and has been predictive of human responses to antiviral drugs [27]. The woodchuck model has been also used to test antitumor compounds for prevention and treatment of HCC [28–31]. HCC develops and is fatal in 100% of woodchucks that are chronically infected with WHV. The median time for HCC appearance in woodchucks is 24 months of age, the median life expectancy is 30 to 32 months, and after identification of HCC the median survival time is six months, a situation similar to patients with HCC [30, 32]. Furthermore, WHV-induced HCC strongly resembles HBV-induced primary liver cancer in humans [30, 32–34]. Comparable to the HCC development process in humans, liver tumors in woodchucks obtain their malignancy in a stepwise process. These distinct characteristics greatly support the preclinical testing of new prophylactic and therapeutic strategies against HBV-induced HCC in woodchucks.

In the present study, antiviral and antitumor efficacy of JVRS-100 was evaluated in chronic WHV carrier woodchucks with pre-existing liver tumors during intravenous (IV) treatment for 12 weeks. Compared to placebo-treated animals, JVRS-100 administration resulted in a reduction of serum viral markers that was associated with the prevention of new liver tumor formation but did not have an apparent effect on established liver tumors.

## Methods

### Preparation of JVRS-100

JVRS-100 was manufactured by Juvaris BioTherapeutics, Inc. (Pleasanton, CA). The compound was prepared by mixing cationic lipid DOTIM (1-[2-(oleoyloxy)ethyl]-2-oleyl-3-(2-hydroxyethyl)imidazolinium chloride) and neutral lipid cholesterol with plasmid DNA (pMB75.6; 4,242 bp in length) in the presence of lactose followed by lyophilization and storage at 2-8 °C. JVRS-100 was reconstituted prior to use by the addition of sterile water for injection for IV administration at the indicated dosage.

### Determination of JVRS-100 mediated immune activation

ELISA-based assays for the detection of woodchuck cytokines and T cell surface markers in blood are not available. For circumventing this limitation, real time RT-PCR-based assays for the detection of mRNA expression of cytokines and T cell surface markers in woodchuck peripheral blood mononuclear cells (PBMCs) and liver were applied as described [31, 35, 36]. Aliquots of PBMCs or liver were lysed using the RNeasy Kit (Qiagen) according to the manufacturer’s specifications and total RNA isolated. RNA was then treated with DNase I (Invitrogen) and reverse transcribed to complementary (c) DNA with MultiScribe Reverse Transcriptase (Applied Biosystems) using oligo(dT). Triplicates of cDNA were amplified by real time PCR on a 7000 Real Time PCR System instrument (Applied Biosystems) using SYBR Green Master Mix (Applied Biosystems) and woodchuck-specific primers for amplification of interferon-alpha (IFN-α), interferon-gamma (IFN-γ), tumor necrosis factor-alpha (TNF-α), IL-2, IL-6, IL-10, IL-12, clusters of differentiation 4 and 8 (CD4 and CD8), and forkhead box P3 (FoxP3) [36, 37]. Target gene expression was normalized *via* the expression of woodchuck β-actin mRNA (PBMCs) or 18S rRNA (liver) [36, 38]. Transcription levels of woodchuck target genes were determined by the formula 2^Δ*Ct*^, where Δ*C*_*t*_ indicates the difference in the threshold cycle between housekeeping and target gene expression. Results were represented as a fold increase of the transcription level in PBMCs or liver obtained from woodchucks following dosing with JVRS-100 relative to animals administered vehicle.

### Antiviral and antitumor efficacy study design

The animal protocol and all procedures involving woodchucks were approved by the Cornell University Institutional Animal Care and Use Committee and adhered to the national guidelines of the Animal Welfare Act, the Guide for the Care and Use of Laboratory, and the American Veterinary Medical Association. Twelve adult woodchucks of either gender, approximately two years of age, seropositive for WHV and with pre-existing liver tumors were used for the evaluation of antiviral and antitumor activity mediated by JVRS-100. These woodchucks were born to WHV-negative females, inoculated at three days of age with a standardized inoculum containing WHV strain 7 (WHV7), and reared in the animal facilities at Cornell University. The chronic WHV carrier status of woodchucks at approximately two years after birth was confirmed serologically by testing for the presence of WHV DNA, WHV surface antigen (WHsAg), and antibodies against WHV core antigen, and for the absence of antibodies against WHsAg (anti-WHs) [39]. Woodchucks for use had at least one hepatic tumor of approximately 1 cm or more in diameter within the left lateral liver lobe as identified by elevated serum activity of gamma-glutamyl transferase (GGT; i.e., > 10 IU/L) and by hepatic ultrasound examination [30]. Characteristically liver tumors of 1 cm or more in diameter are well differentiated or moderately well differentiated trabecular HCCs [30]. One or more ultrasound images were maintained as reference for post-treatment comparisons. The woodchucks were then stratified as they entered the study sequentially into either a JVRS-100 treatment group or a vehicle-treated control group. The initial group of three woodchucks was dosed IV with 100 μg JVRS-100/animal every second week for 12 weeks starting at T_0_, while the control group of three other woodchucks received IV vehicle as placebo at the same time points. An additional three woodchucks were dosed IV with 300 μg JVRS-100/animal every second week for 12 weeks starting at T_0_, while an additional three woodchucks received IV vehicle as placebo at the same time points. For the analysis of antiviral and antitumor effects mediated by JVRS-100, and for simplicity of data presentation, all six placebo-treated animals were included in one group. This study design allowed to compare two JVRS-100 dose groups (*n =* 3/group) with one control group (*n =* 6).

As woodchucks entered the study (T_0_), animals were anesthetized and weighed, and a blood sample was obtained and used for serological testing, for determination of serum WHV DNA loads, and for determinations of clinical chemistry parameters and complete blood counts. JVRS-100 or vehicle was then administered IV using the sublingual vein. Additional blood samples were collected biweekly. Additional ultrasound examinations were performed every second week over a period of 24 weeks. At the indicated time points, all woodchucks were anesthetized, weighed, bled, the liver examined by ultrasound, and JVRS-100 or vehicle administered IV. Hepatic expression of woodchuck cytokines and T cell surface markers was determined in liver biopsy samples collected at pre-treatment, during treatment (week 6), at the end of treatment (week 12), and at the end of the study (week 24). After the completion of the study, all woodchucks were euthanized and complete post-mortem examinations performed.

### Determination of changes in the chronic WHV carrier status of woodchucks

Serum WHV DNA was measured by two different methods depending on concentration: (1) WHV DNA was assayed by dot blot hybridization using three replicate samples of undiluted serum and comparison to a standard dilution series of WHV recombinant DNA plasmid (assay sensitivity, ≥ 1.0x10^7^ WHV ge/ml; or (2) by real time PCR assay of three replicate samples of WHV DNA extracted from 200 μl of serum and comparison to parallel PCR assays of 10-fold dilutions of the WHV DNA plasmid standard (assay sensitivity, ≥ 1.0 × 10^3^ WHV ge/ml) [39]. Levels of WHsAg and of anti-WHs antibodies in serum were determined by ELISA using 1:50 dilutions of serum to insure detection of all markers under saturating conditions [40]. Serum enzyme activities such as GGT, sorbitol dehydrogenase (SDH), alanine aminotransferase (ALT), aspartate aminotransferase (AST), and alkaline phosphatase (ALP), and complete blood counts were assayed as described [39].

### Determination of changes in HCC status of woodchucks

The rate of tumor growth and echoic characteristics of hepatic tumors present before and/or developing during and following treatment with JVRS-100 or vehicle were assessed by ultrasonography. During each ultrasound examination three separate three-dimensional measurements were performed, the diameter recorded, and one or more images recorded digitally for retrospective analysis. Tumor volumes and growth rates of woodchucks in all groups were calculated and compared.

Tumor burden was assessed by examination of digital pictures of the diaphragmatic and visceral surfaces of the liver obtained during post-mortem examination following euthanasia at the end of the study. The total number of tumors and of all other hepatic neoplasms present at necropsy was counted, the mean diameter of each determined by direct caliper measurement, and the total tumor volume (in cubic centimeters) of tumors greater than 1 cm in diameter calculated. The antitumor effect induced by JVRS-100 was then determined by comparing the total number of tumors in the liver and the total tumor volume in woodchucks treated with JVRS-100 at two separate doses to the same parameters of woodchucks that received vehicle-placebo.

### Statistics

The antiviral and antitumor parameters were compared between the groups of woodchucks using Student’s *t*-test. *P* values of < 0.05 were considered statistically significant.

## Results

### Immune responsiveness of woodchucks with increasing viral loads

**Fig. 1:**
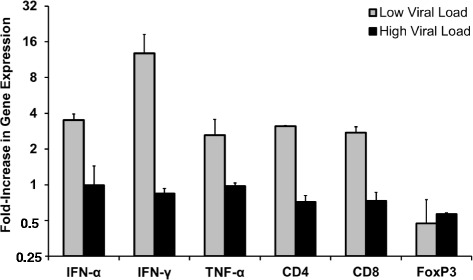
Immune responsiveness of woodchucks with increasing viral loads following a single IV dose of JVRS-100. Fold increase in mRNA expression of cytokines and T cell surface markers in peripheral blood following a single IV dose of JVRS-100 at a concentration of 50 or 100 μg/animal into woodchucks with low (*n =* 2) *versus* high (*n =* 2) serum WHV DNA loads. Results are presented as a change from the transcription level observed in control woodchucks with comparable low (*n =* 2) or high (*n =* 2) viral loads following a single IV dose of placebo. Vertical lines denote standard deviations

**Fig. 2:**
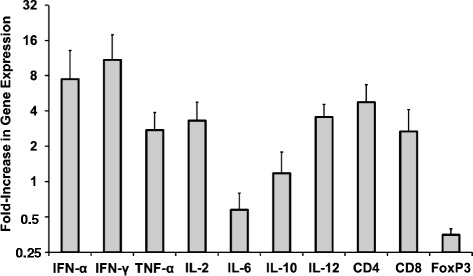
Immune responsiveness of woodchucks with low viral loads following a single IV dose of JVRS-100. Fold increase in mRNA expression of cytokines and T cell surface markers in peripheral blood following a single IV dose of JVRS-100 at a concentration of 100 μg/animal into woodchucks with low serum WHV DNA load (*n =* 4). Results are presented as a change from the transcription level observed in control woodchucks with comparable low viral load following a single IV dose of placebo (*n =* 4). Vertical lines denote standard deviations

For determining the dependency of responsiveness to immune stimulation on serum WHV DNA, cytokine and T cell surface marker mRNA expression was evaluated following dosing of JVRS-100 in four age- and gender-matched chronic WHV carrier woodchucks with low (mean: 2.5 × 10^10^ genomic equivalents (ge)/ml) *versus* high (mean: 6.0 × 10^11^ ge/ml) viral load. The working hypothesis was that high viral load that is usually seen in chronic WHV infection (approximately 100-fold higher than the typical human viral load) results in immune suppression to external immune stimuli. Woodchucks were dosed IV once with JVRS-100 (concentration range: 50 to 100 μg/animal) and then evaluated for mRNA expression of important antiviral cytokines, such as IFN-α, IFN-γ, and TNF-alpha, and for T cell surface markers, such as CD4 and CD8 in PBMCs obtained at eight hours post-injection. FoxP3 expression was included in this analysis for determining changes in regulatory T (T_reg_) cell function. The single dose of 50 to 100 μg/animal applied to woodchucks was based on IV dosing in mice where it has been established that 1 to 10 μg/animal elicited a robust cytokine response [21]. Since interspecies differences in the response to JVRS-100 are known (personnel communication; J. Fairman) and data on the use of this immunostimulant in woodchucks were lacking, it was decided to start dosing at the low end of the activity range. Age- and gender-matched control woodchucks with comparable low or high viral load were dosed IV once with placebo and the expression of cytokines and T cell surface markers also determined at eight hours post-injection. Relative to placebo administration in control animals, the woodchucks with low serum viral load showed an increase in the expression of all markers with the exception of FoxP3, which was slightly down-regulated following administration of JVRS-100 (Fig. 1). In contrast, and again relative to placebo administration in control animals, the woodchucks with high serum viral load demonstrated little change in cytokine or T cell surface marker expression (Fig. 1), suggesting a general unresponsiveness to stimulation with JVRS-100 at the selected dose range. For verifying the JVRS-100 mediated response in woodchucks with low viremia with a broader panel of cytokines, and for differentiating between T_H_1 and T_H_2 cellular immune responses, four additional age-matched, female chronic WHV carrier woodchucks with low serum WHV DNA (mean: 1.3 × 10^10^ ge/ml) were dosed IV once with 100 μg JVRS-100 and the expression of the above markers, in addition to the cytokines IL-2, IL-6, IL-10, and IL-12, was evaluated at eight hours post-injection (Fig. 2). Age- and gender-matched control animals with comparable low viral load were dosed IV once with placebo and the expression of all markers was also determined at eight hours post-injection. Relative to placebo administration in control animals, all woodchucks that received JVRS-100 demonstrated a marked upregulation of the expression of T cell surface markers and of mainly T_H_1 cytokines, such as IFN-α, IFN-γ, TNF-α, IL-2, and IL-12. Regarding T_H_2 cytokines, minimal changes were observed in the expression of IL-10, while IL-6 and the T_reg_ marker FoxP3 were slightly down-regulated. Considering the observed responsiveness in these animals that suggested a T_H_1 skew toward cellular immune responses following JVRS-100 administration, the follow-on antiviral and antitumor study was conducted in woodchucks which had low serum WHV DNA of 2.0 × 10^10^ ge/ml on average at the start of treatment, which is still higher but more comparable to the viral load typically seen in HBV-infected patients.

### In vivo antiviral and antitumor effects mediated by JVRS-100 in chronic WHV infected woodchucks with HCC

**Fig. 3:**
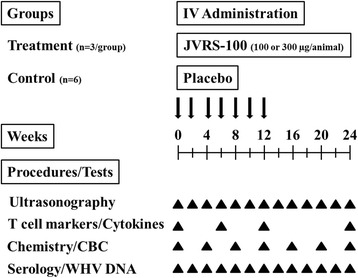
Experimental outline for IV administration of JVRS-100 in woodchucks with pre-existing liver tumors for determining antiviral and antitumor effects. CBC: Complete blood counts

Before woodchucks entered the study, all animals had been confirmed as chronic WHV carriers based on the presence of WHV DNA and WHsAg and absence of anti-WHs in serum. All woodchucks had (sometimes highly) elevated serum levels of GGT (range: 12–203 IU/L), which is an oncogenic biomarker in this animal model and indicative of pre-existing liver tumors that were also confirmed by ultrasonography. Animals presented with 1–3 tumors at this time and the average tumor size ranged between 0.93 and 5.03 cm in diameter. JVRS-100 or vehicle was then administered IV at T_0_ using the sublingual vein (Fig. 3). Administrations were repeated every 2 weeks thereafter for a total of 12 weeks (i.e., 7 doses of JVRS-100 or vehicle placebo were given). After the end of treatment, animals were followed for additional 12 weeks. Blood sample collection for serological testing and ultrasound examinations for determinations of tumor growth and new tumor formation were performed biweekly, while liver tissues for expression analysis of cytokines and T cell surface markers were collected at pre-treatment, and again at weeks 6, 12 and 24 (Fig. 3).

Following an initial group of three woodchucks dosed with 100 μg JVRS-100 and of three other woodchucks administered vehicle as placebo control, an additional three woodchucks were dosed with 300 μg JVRS-100 and controlled by three other placebo-treated woodchucks. This study design allowed to compare two dose groups (*n =* 3/group) with one control group (*n =* 6). There were no flare reactions during or following treatment with JVRS-100 as determined by the changes in serum activity of liver enzymes, such as SDH, ALT, AST, and ALP (data not shown). Occasionally, normalization of ALP and AST levels, and in part also of ALT and GGT levels, was observed during treatment with JVRS-100 mainly at the higher dose; however, most liver enzymes including GGT rose again after the end of treatment.

**Fig. 4:**
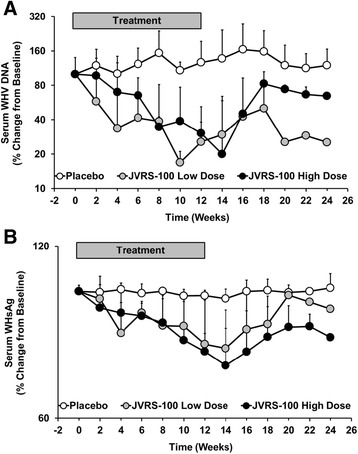
Antiviral effects mediated by JVRS-100 in chronic WHV infected woodchucks with HCC. Percentage change in serum WHV DNA (**a**) and WHsAg (**b**) from pre-treatment level at T_0_ in woodchucks with pre-existing liver tumors during and following IV treatment with JVRS-100 at concentrations of 100 μg/animal (low dose; *n =* 3) or 300 μg/animal (high dose; *n =* 3) or with vehicle (placebo; *n =* 6) for 12 weeks. Vertical lines denote standard deviations

Regarding antiviral effects, all woodchucks treated with JVRS-100 demonstrated reductions in serum WHV DNA and WHsAg during treatment. When normalized for the differing amounts of initial viremia and antigenemia, there was a steady decrease in the treated animals when compared with the control animals, and the decline in WHV DNA was more pronounced than for WHsAg (Fig. 4). In addition, woodchucks treated with the higher dose of JVRS-100 showed significant reductions (*P* < 0.05) in serum WHV DNA (weeks 2, 4, 8–16, and 20–24) and WHsAg (weeks 2–24) when the group mean was compared to that of the control group. Less notable reductions were observed for woodchucks treated with the lower dose of JVRS-100 but the decline in serum WHV DNA was statistically significant (*P* < 0.05) at week 10. Despite these declines in viremia and antigenemia, none of the JVRS-100 treated woodchucks demonstrated seroconversion to anti-WHs antibodies (data not shown). Antiviral effects were transient as serum WHV DNA and WHsAg started to relapse during weeks 14–16 after the cessation of JVRS-100 treatment, although levels of viremia and antigenemia stayed below those of the control group during most time points of the follow-up period.

**Fig. 5:**
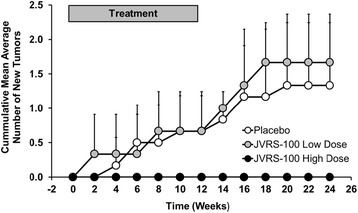
Antitumor effects mediated by JVRS-100 in chronic WHV infected woodchucks with HCC. Cumulative mean average number of new liver tumors developed in woodchucks with pre-existing liver cancer during and following IV treatment with JVRS-100 at concentrations of 100 μg/animal (low dose; *n =* 3) or 300 μg/animal (high dose; *n =* 3) or with vehicle (placebo; *n =* 6) for 12 weeks. Vertical lines denote standard deviations

Regarding antitumor effects, there was no difference in the tumor burden as measured by the total volume of pre-existing liver tumors in woodchucks during and following treatment with JVRS-100 at both doses when compared to placebo-treated animals (data not shown), suggesting that JVRS-100 based immunotherapy had no apparent effect on already established tumors. However, there was a difference in the formation of additional tumors following initiation of treatment with JVRS-100 at the high dose. As shown in Fig. 5, the cumulative mean average number of new liver tumors detected in woodchucks was comparable (*P* > 0.05) between the six animals that received placebo and the three animals administered the low dose of JVRS-100. This was in clear contrast to the three woodchucks treated with the high dose of JVRS-100 in which new liver tumors did not develop over the 24 weeks of the study as determined by ultrasound examination and as confirmed at necropsy, suggesting that JVRS-100 at a well-tolerated dose of 300 μg/animal mediated immune effects that prevented tumor spread and metastasis. The prevention of new liver tumors was significant (*P* < 0.05) from week 16 onward when the cumulative mean average number was compared to woodchucks receiving JVRS-100 at the lower dose of 100 μg/animal. Although the number of animals in the treatment groups were limited, this finding is highly unusual for this disease model in which woodchucks with pre-existing liver tumors have a survival time of six months before they die or need to be euthanized due to seizures known to be associated with the development of terminal HCC. The above finding, therefore, may present an important discovery for further development of immunotherapy as an intervention for HBV-induced HCC that need to be investigated in more detail.

**Fig. 6:**
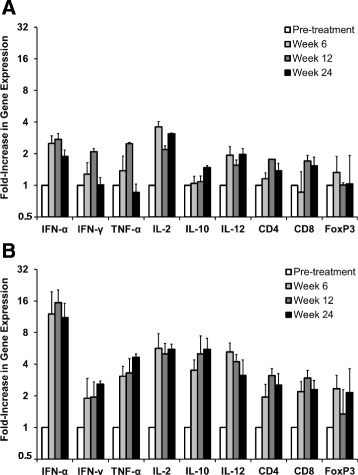
Intrahepatic expression of cytokines and T cell surface markers mediated by JVRS-100 in chronic WHV infected woodchucks with HCC. Fold increase in mRNA expression of cytokines and T cell surface markers in liver of woodchucks with pre-existing liver cancer during and following IV treatment with JVRS-100 at concentrations of 100 μg/animal (low dose; *n =* 3) (**a**) or 300 μg/animal (high dose; *n =* 3) (**b**). Results are presented as a change from the transcription level observed in control woodchucks during and following IV treatment with vehicle (placebo; *n =* 6). Vertical lines denote standard deviations

Regarding the expression of cytokines and T cell surface markers in liver, woodchucks treated with the higher dose of JVRS-100 demonstrated increases in CD4 and CD8 and in mainly T_H_1 cytokines such as IFN-α, TNF-α, IL-2, and IL-12 during and following treatment relative to control animals (Fig. 6). The expression of the T_H_2 cytokine IL-10 was also increased as well as was the expression of the T_reg_ marker FoxP3 although to a lesser degree. Since the magnitude and duration of expression of CD4, CD8, IFN-α, TNF-α, and IL-12 was less pronounced in woodchucks treated with the lower dose of JVRS-100, the overall results suggest that administration of JVRS-100 at an effective and safe dose activates an antiviral and antitumor immunity that is mainly mediated by the induction of T_H_1 immune responses in liver and periphery and thereby blocks the conversion of viral-induced chronic liver disease into HCC in vivo.

## Discussion

The recent development of effective nucleos(t)ide analogs with high barrier to viral resistance represents substantial progress in the control of chronic HBV infection. However, treatment of chronic hepatitis B (CHB) is still challenging as these direct acting antivirals do not target the viral, covalently-closed circular (ccc) DNA molecule within the nucleus of hepatocytes, which is representing the HBV genome and that is utilized by the virus as a template for synthesizing the pre-genomic RNA required for replication [41]. Thus, although the levels of viremia (and of antigenemia to varying degree) are strongly suppressed by treatment with nucleos(t)ide analogs, low-level, residual viral replication persists and supports the maintenance of immune tolerance to chronic HBV infection and associated liver disease progression, including liver tumor formation and HCC. As recrudescence of viral replication is frequently observed following cessation of treatment with nucleos(t)ide analogs [9], prolonged or even lifelong therapy is needed to produce sustained control of HBV infection. Unlike (pegylated) IFN-α, which has inhibitory effects on HBV replication and transcription, nucleos(t)ide analogs do not induce broad immunostimulatory activity that facilitates immune clearance of HBV-infected hepatocytes [42]. IFN-α has many immunostimulatory properties such as activation of innate and adaptive immune responses against HBV but its use as an anti-HBV therapeutic is limited by the severe toxicity observed in the majority of treated patients [43]. Thus, continued development of new immunostimulatory compounds against HBV is warranted that can mimic the benefits of IFN-α without its toxicity. In the present study, the antiviral and antitumor properties of JVRS-100, a potent stimulant of innate immunity [17, 18, 20], were tested in woodchucks with chronic WHV infection and pre-existing liver tumors.

Chronic WHV infection in woodchucks closely reproduces the virological, immunological, and histopathological features observed in chronic HBV infection in humans [26]. An important difference is that chronic WHV infection is associated with a considerably higher viral load which frequently exceeds 10^10^ ge/ml. In human CHB, patients classified as being high viremic have maximum HBV DNA serum concentrations of approximately 10^8^ ge/ml. These high levels of circulating WHV virions have been implicated in the impairment of immune response in woodchucks, including exhaustion of effector T cell function [44, 45], thereby rendering chronic WHV infection into a disease condition that is extremely difficult to treat. As shown in the present study, responsiveness to immune stimulation with a single dose of JVRS-100 demonstrated a dependency on viral load as only woodchucks with relatively low viremia level had an increase in transcripts for antiviral cytokines and T cell surface markers in peripheral blood (Fig. 1 and Fig. 2). In line with previous results in WHV-naïve woodchucks [35] and other animal models [17, 24], JVRS-100 administration to chronic WHV carrier woodchucks changed the T_H_1/T_H_2 balance, with an apparent TH_1_ skew toward cellular immune responses as suggested by the upregulated expression of IFN-α, IFN-γ, TNF-α, IL-2, and IL-12 (Fig. 2).

Based on the above findings, chronic WHV carrier woodchucks with low viremia were subsequently selected for the antiviral and antitumor efficacy study (Fig. 3). Repeated administration of JVRS-100 for 12 weeks induced marked but transient antiviral effects, including declines in serum WHV DNA and WHsAg that were more pronounced in woodchucks treated with the higher dose and that stayed suppressed for most time points during the follow-up period (Fig. 4). These findings in woodchucks are in line with other studies demonstrating that immunotherapy in the setting of low viremia is antiviral efficacious whereas in the setting of high viremia, the same therapeutic approach leads to an activation of immunosuppressive mechanisms thereby abolishing the antiviral effect of treatment. For example, high viral and/or antigen load have been implicated to be an important cause of T cell hyporesponsiveness to HBV antigens [46, 47]. In addition, high viremia has been shown to be a main factor that negatively predicts antiviral response to IFN-α treatment in patients with chronic HBV infection [48]. In woodchucks, hepatic delivery of the immunostimulatory cytokines IL-12 or IFN-α fused to apolipoprotein A-I by viral vectors resulted in marked antiviral effects [36, 49], but only in animals with relatively low viral load (below 10^10^ ge/ml) which was not observed in animals with high viremia (≥10^11^ ge/ml). Furthermore, woodchucks with response to IL-12 therapy developed cellular immune responses against WHV antigens and had a decrease in T_reg_ cells such as FoxP3-expressing cells [36]. Contrary, high-viremic woodchucks unresponsive to IL-12 therapy had a significant increase in FoxP3 expression and failed to develop WHV-specific cellular responses [36]. In regard to T_reg_ cells, JVRS-100 in the present study was found to have little impact on the expression of FoxP3 at eight hours post-injection, and a single dose administration of JVRS-100 did not result in apparent expression differences for FoxP3 in the setting of low *versus* high viremia (Fig. 1 and Fig. 2). Other molecules or cytokines with inhibitory functions such as programmed death 1 (PD-1), its ligand (PD-L1), and transforming growth factor beta (TGF-β) that were not tested in the present study may play a role in the observed unresponsiveness of high-viremic woodchucks to JVRS-100. Overall, the peripheral blood system and the liver of woodchucks with high viremia appear to present a highly suppressive environment that can counter-regulate antiviral effects induced by immunotherapy with broad-acting compounds such as IL-12 [50], IFN-α [49] or JVRS-100. This appears opposite to immunotherapy with specific-acting compounds such as the small molecule TLR7 agonist GS9620 and the viral sensor protein activator SB 9200 that induced pronounced and sometimes sustained antiviral effects in woodchucks with high viremia [38, 51, 52]. Thus, it is important to note for further immunotherapeutic development in the woodchuck model that animals with relatively low viremia responded to JVRS-100, whereas animals with relatively high viremia appeared unresponsive. This may have important implications in the selection of potential patients with CHB for treatment with JVRS-100 in a future clinical trial.

Aside from the immunostimulatory activity in the setting of low *versus* high viremia, the antiviral response induced by JVRS-100 in the present study (Fig. 4) was in the range of those of nucleos(t)ide analogs previously evaluated in the woodchuck model. The magnitude of viral load reduction with JVRS-100, especially at the high dose, was comparable to lamivudine and emtricitabine after administration for 12 weeks [39]. Common for these compounds but somewhat different to JVRS-100 was the immediate rebound of WHV markers following cessation of treatment. Comparable to JVRS-100, these nucleos(t)ide analogs also induced minor, transient increases in liver enzymes during treatment, and before serum activity of AST, ALP and/or ALT became normalized. As elevations in these liver enzymes noted during JVRS-100 treatment at week 8 were temporally associated with the reductions in serum WHV DNA (and WHsAg), their rise may indicate immune-mediated viral clearance of infected hepatocytes by CTLs and NK cells, as also observed in other studies for this compound [20–22]. As liver enzyme activity was transient and became normalized at the end of treatment, this may further indicate that other, non-cytolytic mechanism(s) contributed to the suppression of WHV replication. The data in chronic WHV carrier woodchucks is also in agreement with the demonstrated efficacy of JVRS-100 in HBV transgenic mice. In a dose-ranging study in this animal model, IV administration of JVRS-100 (0.005 to 5 μg/mouse) on days 1, 7, and 13 resulted in significant reduction in liver HBV DNA after 14 days and the achieved antiviral effect at higher doses was comparable to that of adefovir [53]. Suppression of hepatic HBV DNA in mice by JVRS-100 was associated with significantly increased cytokine levels in liver and serum, including the T_H_1 cytokine IL-12. This result was confirmed by the current study since elevated transcript levels of CD4 and CD8 and of mainly T_H_1 cytokines (IFN-α, TNF-α, IL-2, and IL-12) were observed in woodchucks that were treated with the higher dose of JVRS-100 and which demonstrated more pronounced antiviral and antitumor effects when compared to animals treated with the lower dose (Fig. 6). However, expression of IL-10, an immunosuppressive cytokine produced by T_reg_ cells and various other cells, was markedly upregulated by the high dose of JVRS-100, which was consistent with the increase in FoxP3 expression.

Treatment of chronic WHV carrier woodchucks without pre-existing liver tumors at pre-treatment with nucleos(t)ide analogs and immunostimulators has been shown to delay or even prevent the onset of HCC development [30, 51]. The conclusion from these studies is that prolonged suppression of viral replication results in less liver injury and cellular damage thereby deferring transformation of altered hepatocytes into tumors. The effect of the above treatment on pre-existing liver tumors, however, is unknown as drug efficacy studies in the woodchuck are typically initiated in HCC-free animals as determined by ultrasonography and low GGT levels. Studies which tested antitumor treatment of pre-existing liver tumors in woodchucks by delivery of murine IL-12, alone or in combination with the costimulatory factor B7.1, demonstrated partial remission based on transient changes in tumor growth [29, 31]. However, these studies did not evaluate if formation of new tumors was abolished as observed in the present study (Fig. 5). The antitumor response in woodchucks of the above studies [29, 31] as well as in the current study was associated with a general activation of cellular and/or hepatic immune responses, including the induction of T_H_1 cytokines and T cell markers. As this response is comparable to that mediated by JVRS-100 in another animal model of cancer [25] and in woodchucks with low viremia and/or pre-existing liver tumors (Fig. 1, Fig. 2 and Fig. 6), it is tempting to speculate that dosing with JVRS-100 at an increased frequency and/or at a higher dose may have resulted in direct antitumor effects that needs to be explored in a future study. However, the finding that JVRS-100 at a high dose mediates an effect on the formation of new tumors has not been demonstrated for other compounds in animal models of virus-induced HCC.

## Conclusions

Since treatment with JVRS-100 was effective and safe in preventing new liver tumor formation in woodchucks with chronic WHV infection and established HCC, it deserves consideration as a potential therapy for patients with CHB, especially in patients who have low HBV load or who are at a higher risk for development of HBV-induced HCC.

## Abbreviations

ALP: Alkaline phosphatase
ALT: Alanine aminotransferase
Anti-WHs: Antibodies against WHsAg
AST: Aspartate aminotransferase
cccDNA: Covalently-closed circular DNA
CD: Clusters of differentiation
cDNA: Complementary DNA
CHB: Chronic hepatitis B
CTL: Cytolytic T lymphocytes
DOTIM: (1-[2-(oleoyloxy)ethyl]-2-oleyl-3-(2-hydroxyethyl)imidazolinium chloride
FoxP3: Forkhead box P3
Ge: Genomic equivalents
GGT: Gamma-glutamyl transferase
HBV: Hepatitis B virus
HCC: Hepatocellular carcinoma
IFN: Interferon
IL: Interleukin
IV: Intravenous
JVRS-100 or CLDC: Complexes of cationic/neutral lipid carrier and non-coding DNA plasmid
NK: Natural killer cells
PBMCs: Peripheral blood mononuclear cells
PD-1: Programmed death receptor
PD-L1: Programmed death receptor 1 ligand
SDH: Sorbitol dehydrogenase
TGF: Transforming growth factor
T_H_: T helper cells
TLR: Toll-like receptor
TNF: Tumor necrosis factor
T_reg_ cells: Regulatory T cells
WHsAg: WHV surface antigen
WHV: Woodchuck hepatitis virus
